# Longitudinal RNA-Seq analysis of acute and chronic neurogenic skeletal muscle atrophy

**DOI:** 10.1038/s41597-019-0185-4

**Published:** 2019-09-24

**Authors:** Jeffrey T. Ehmsen, Riki Kawaguchi, Ruifa Mi, Giovanni Coppola, Ahmet Höke

**Affiliations:** 1 Department of Neurology, Neuromuscular Division, Johns Hopkins School of Medicine, Baltimore, MD 21205 USA; 2 Department of Neurology, University of California, Los Angeles, Los Angeles, CA 90095 USA; 3 Department of Psychiatry and Biobehavioral Sciences, University of California, Los Angeles, Los Angeles, CA 90095 USA

**Keywords:** Somatic system, Regeneration and repair in the nervous system

## Abstract

Skeletal muscle is a highly adaptable tissue capable of changes in size, contractility, and metabolism according to functional demands. Atrophy is a decline in mass and strength caused by pathologic loss of myofibrillar proteins, and can result from disuse, aging, or denervation caused by injury or peripheral nerve disorders. We provide a high-quality longitudinal RNA-Seq dataset of skeletal muscle from a cohort of adult C57BL/6J male mice subjected to tibial nerve denervation for 0 (baseline), 1, 3, 7, 14, 30, or 90 days. Using an unbiased genomics approach to identify gene expression changes across the entire longitudinal course of muscle atrophy affords the opportunity to (1) establish acute responses to denervation, (2) detect pathways that mediate rapid loss of muscle mass within the first week after denervation, and (3) capture the molecular phenotype of chronically atrophied muscle at a stage when it is largely resistant to recovery.

## Abstract (summary)

**Table:** 

Measurement(s)	skeletal muscle atrophy • gene expression
Technology Type(s)	RNA sequencing
Factor Type(s)	denervation status • denervation duration
Sample Characteristic - Organism	Mus musculus

Machine-accessible metadata file describing the reported data: 10.6084/m9.figshare.9751892

## Background & Summary

Skeletal muscle atrophy is the loss of muscle mass and function that occurs in response to diverse stimuli including disuse/immobility, glucocorticoid treatment, cancer, aging, and denervation^1–5^. Biologically, atrophy reflects the active loss of skeletal muscle contractile proteins, leading to loss of strength and functional impairment with substantial impact on quality of life and, in some cases, reduced survival^6–8^. In addition, chronically denervated, atrophied muscle shows impaired capacity for reinnervation and functional recovery, which significantly limits prospects for recovery in settings of chronic neuromuscular disease, delayed repair, or large nerve lesions^9–12^.

Nerve-evoked contraction is the most important factor for maintaining or regaining muscle mass and force^13^. Neurogenic atrophy refers specifically to skeletal muscle atrophy resulting from denervation, as may occur in traumatic injury or diseases that affect the peripheral nervous system, such as amyotrophic lateral sclerosis (ALS)^14–17^. A number of “atrogenes” are induced as a result of denervation and in response to various triggers of muscle atrophy; among these are specific ubiquitin ligases targeting components of the sarcomere^18–29^. A comprehensive analysis of the global gene pathways that change in response to denervation and during atrophy may offer an optimal chance of identifying means to pharmacologically maintain or increase muscle mass and function in atrophy-associated disease states.

We provide here a comprehensive RNA-Seq dataset^30^ to identify gene expression changes across the entire longitudinal course of muscle atrophy, affording the opportunity to (1) establish acute responses to denervation within the first day, (2) detect pathways that mediate rapid proteolysis and loss of muscle mass within the first week after denervation, and (3) capture the molecular phenotype of chronically atrophied muscle (weeks to months after denervation) at a stage when it is largely resistant to reinnervation and recovery.

**Fig. 1:**
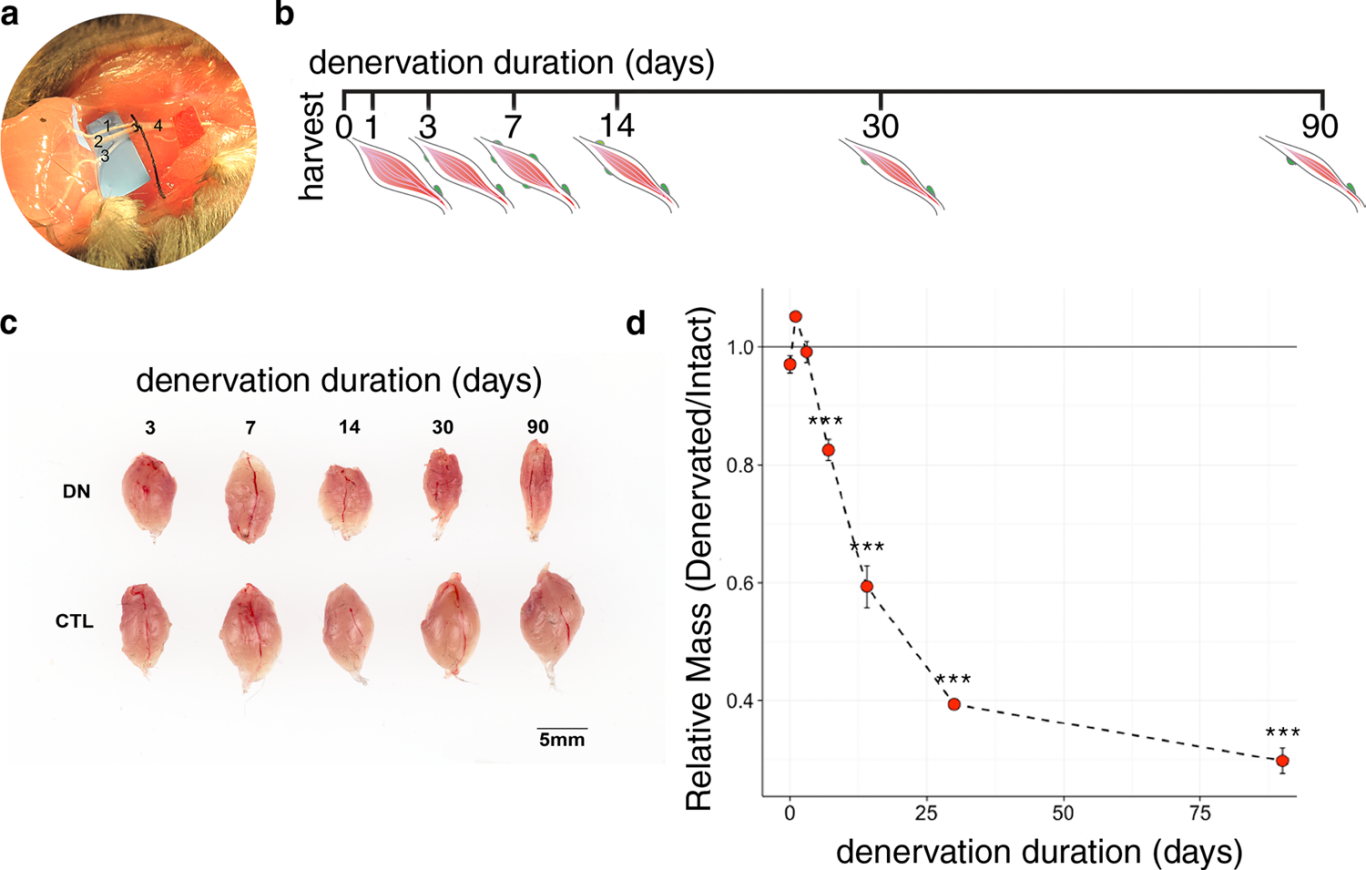
Overview of the experimental procedure. The tibial nerve, the largest branch of the sciatic nerve, supplies the gastrocnemius muscle and other muscles of the lower limb posterior compartment. In our mouse model of denervation atrophy, the sciatic nerve is identified, and its branches separated to isolate the tibial nerve (**a**; nerve identities are as follows: 1, sural nerve; 2, tibial nerve; 3, common peroneal/fibular nerve; 4, sciatic nerve). We generated a cohort of C57BL/6 J male mice denervated for 0, 1, 3, 7, 14, 30, or 90 days (**b,c**). Significant atrophy is apparent by 7 days after denervation, with consistent decline in mass during chronic denervation (**d**); ***P < 0.001 compared to baseline.

We generated a longitudinal RNA-Seq dataset from a cohort of adult (8-week-old) wild type C57BL/6 J male mice denervated for 0 (baseline), 1, 3, 7, 14, 30, or 90 days (n = 4 for each timepoint)^30^. We elected to use tibial nerve transection as a model for muscle denervation, as this approach is physiologically meaningful while limiting the morbidity (i.e., pain and immobility) associated with complete sciatic nerve transection^31^. The tibial nerve is the largest branch of the sciatic nerve that supplies skeletal muscles of the posterior compartment of the lower limb, including the gastrocnemius and soleus. In brief, we identified and separated the tibial nerve from other branches of the sciatic nerve, then ligated, cut distally, and sutured the proximal stump in place to prevent muscle reinnervation during chronic studies. We have established that this model reliably induces significant gastrocnemius atrophy within one week after denervation, with atrophy becoming progressively more severe over time (Fig. 1c,d).

The samples collected and described in this manuscript include transcriptional profiles from a total of 28 denervated gastrocnemii and 28 contralateral (paired) intact gastrocnemii, comprising 4 denervated and 4 contralateral (paired) intact gastrocnemii for each of 7 denervation durations [0 (baseline), 1, 3, 7, 14, 30, and 90 days]^30^. All specimens were generated from a cohort of male C57BL/6 J mice that were 8 weeks of age at the start of the study. These data provide a comprehensive description of baseline gene expression in adult mouse skeletal muscle and a broad assessment of the acute and longitudinal gene expression changes in atrophying muscle associated with denervation.

## Methods

### Animal husbandry

8-week-old C57BL/6 J male mice (Stock #000664) were obtained from the Jackson Laboratory (Bar Harbor, ME) and randomized into 7 groups of n = 4 mice per group for the following denervation timepoints: 0, 1, 3, 7, 14, 30, and 90 days. Animal subjects were housed in a controlled environment with a 12:12-h light-dark cycle with ad libitum access to water and food (Envigo 2018 SX). All mouse experiments were carried out under protocols approved by the JHU Animal Care and Use Committee.

### Tibial nerve denervation surgery

Mice were anesthetized with 1.5% isoflurane/2% oxygen using a VetEquip inhalation system (Livermore, CA). The left hindlimb was shaved and sterilized, and a 1 cm incision was introduced in the skin overlying the dorsal thigh. Myofascial planes were gently separated to reveal the sciatic nerve. The tibial nerve branch was identified at its distal branch point and gently separated from the sciatic and peroneal nerves, then ligated proximally and distally using a 10-0 polyamide monofilament suture. The tibial nerve was then transected, the nerve length between ligatures carefully resected, and the proximal stump sutured to the biceps femoris muscle to prevent distal reinnervation. The incision was then closed using stainless steel wound clips. Mice were monitored for recovery from anesthesia and then returned to their home cages.

### Myofiber morphometry

Gastrocnemii were frozen in O.C.T. in liquid nitrogen-cooled isopentane, then sectioned at 10 μm. Mid-belly transverse sections were blocked with M.O.M. in PBS (1:40 dilution, Vector Laboratories, catalogue #MKB-2213) at room temperature for 1 h, then incubated overnight at 4 °C with a mixture of BA-D5 supernatant (1:100, myosin heavy chain type I, SC-71 supernatant (1:100, myosin heavy chain type IIa), BF-F3 concentrate (1:100, myosin heavy chain type IIb) [all from the Developmental Studies Hybridoma Bank (DSHB)], and rat-anti-laminin (1:1000, Sigma, catalogue #L0663) in 1% BSA/PBS. Sections were then washed 3 × 5 min in PBS and incubated with a mixture of the following secondary antibodies (all at 1:500) for 2 h at room temperature: goat-anti-mouse IgG2b-DyLight-405, IgG1-Alexa Fluor-488, IgM-Alexa Fluor-594 (all from Jackson ImmunoResearch, catalogue numbers 115-475-207, 115-545-205, and 115-585-075, respectively), and goat anti-rat-IgG-Alexa Fluor-647 (Thermo Fisher Scientific, catalogue #A-21247), diluted in 1% BSA/PBS. Sections were washed 3 × 5 min in PBS and coverslipped using Prolong Gold antifade (Thermo Fisher Scientific, catalogue #P36930). Transverse sections were imaged in their entirety using a Zeiss AxioObserver. Myofiber minimum Feret diameters were determined using Fiji (NIH)^32^, with ~100 randomly selected myofibers of each fiber type (type I, II, or IIa) measured from each of 3 biological replicates for each indicated timepoint. Statistical analysis was performed using Stata v. 11.2 (College Station, TX)^33^.

### RNA Isolation

Skeletal muscle was homogenized in TRIzol (Ambion, catalogue #15596018) using RNase-free stainless steel beads (Next Advance, catalogue #SSB02-RNA). Homogenates were centrifuged at 10,000 rpm at 4 °C for 10 min to pellet debris, and RNA was purified from the TRIzol supernatant using a Direct-Zol RNA mini purification kit with on-column DNase digestion (Zymo Research, catalogue #R2072). RNA integrity (RIN) was assayed using an Agilent 2100 Bioanalyzer.

### RNA-Seq library preparation, sequencing, and bioinformatics analysis

RNA-sequencing was carried out using TrueSeq RiboZero gold (stranded) kit (Illumina, catalogue #20020597). Libraries were indexed and sequenced over 18 lanes using HiSeq4000 (Illumina) with 69-bp paired end reads. Quality control (QC) was performed on base qualities and nucleotide composition of sequences using FastQC version 0.11.5^34^, to identify problems in library preparation or sequencing. Sequence quality for the dataset described here was sufficient that no reads were trimmed or filtered before input to the alignment stage. Paired-end reads were aligned to the most recent *Mus musculus* mm10 reference genome (GRCm38.75) using the STAR spliced read aligner (version 2.4.0)^35^. Average input read counts were 58.0 M per sample (range 39.1 M to 91.0 M) and average percentage of uniquely aligned reads was 81.9% (range 72.3% to 88.6%). Total counts of read-fragments aligned to known gene regions within the mouse (mm10) refSeq (refFlat version 07.24.14) reference annotation were used as the basis for quantification of gene expression. Fragment counts were derived using HTSeq (version 0.6.0) and the mm10 refSeq transcript model^36^. Low count transcripts were filtered, and count data were normalized using the method of trimmed mean of M-values (TMM)^37^ followed by removing unwanted variation using Bioconductor package RUVseq^38^ with k value of 1. Differentially expressed genes (FDR < 0.1) were then identified using the Bioconductor package limma with voom function to estimate mean-variance relationship, followed by empirical Bayes moderation^39–41^. Pairwise comparisons between denervated and contralateral intact muscle at each timepoint were used as the basis for model contrasts. All bioinformatics analyses were conducted using R version 3.5.1^42^.

## Data Records

Sequencing data in the fastq format have been deposited in NCBI Sequence Read Archive (SRA)^30^. A metadata table (Supplementary Table S1) is available with details for each sample.

## Technical Validation

### Reproducible skeletal muscle atrophy using tibial nerve denervation model

**Fig. 2:**
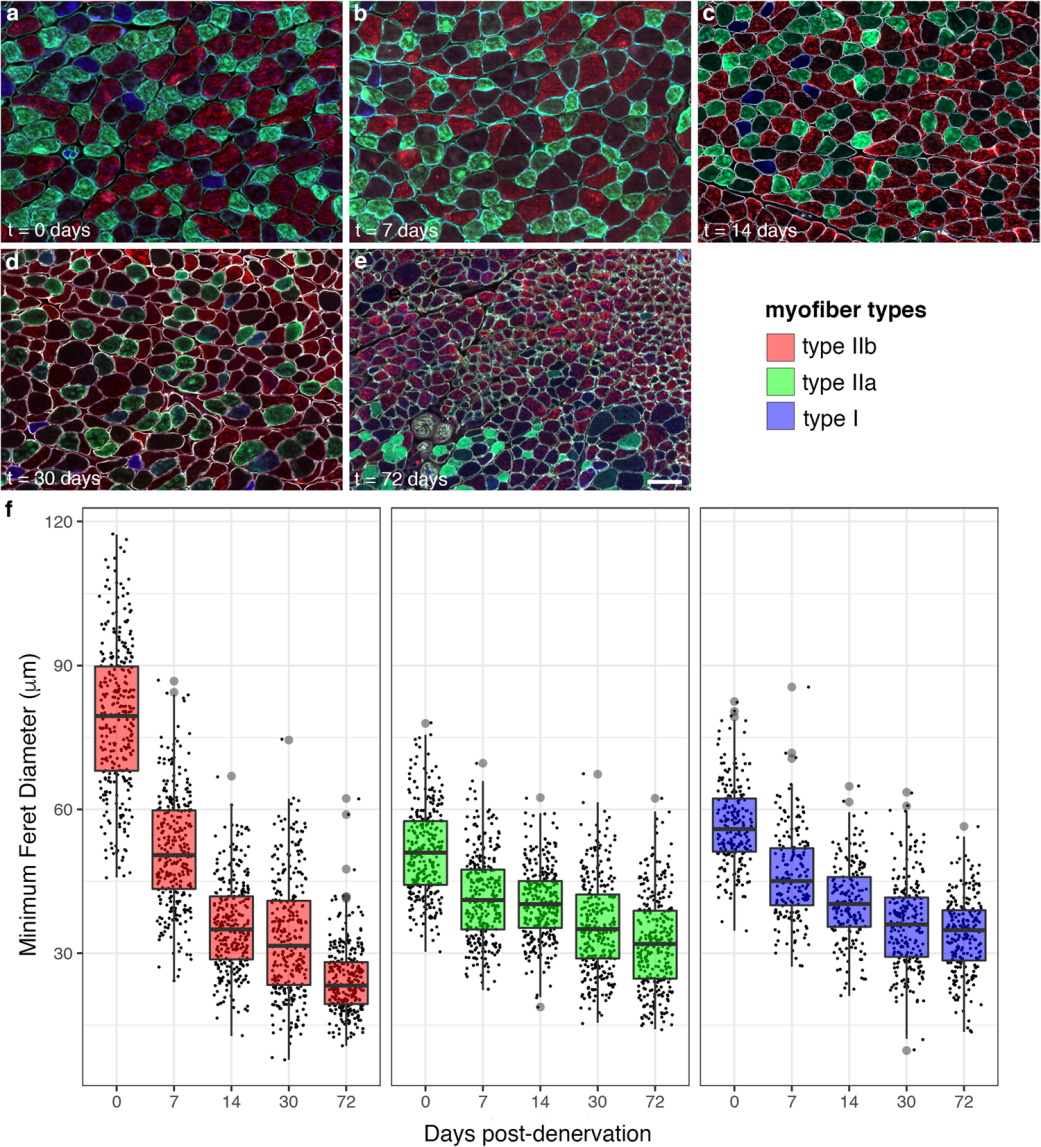
Gastrocnemius myofiber morphometry. Atrophy of type I, IIa, and IIb myofibers was analyzed by assessment of minimum Feret diameter at baseline (t = 0 days) and 7, 14, 30, and 72 days post-denervation. All three myofiber types showed significant atrophy within the first week after denervation, with the greatest change in magnitude observed for type IIb myofibers overall. Scale bar, 100 μm.

Tibial nerve denervation resulted in a reliable time-dependent loss of skeletal muscle mass, with a significant difference in mass between denervated and contralateral intact gastrocnemii detected by day 7 post-denervation (Fig. 1c,d). All mice used in this study entered the cohort at the same time, with sequential denervation according to the designated timepoints, to remove age as a potential confounding variable. Mouse gastrocnemius contains a mixed population of myofiber types including so-called slow twitch myofibers (type I) and fast twitch myofibers (type IIa and IIb). After muscle denervation, all three of these myofiber populations showed a significant reduction in size as measured by minimum Feret diameter, with the most substantial rate of individual myofiber atrophy occurring within the first two weeks post-denervation (Fig. 2). Type IIb myofibers, the most abundant myofiber type in mouse gastrocnemius, showed the largest magnitude of atrophy (Fig. 2f). Multiple linear regression with myofiber type, myofiber type-time interactions, and time modeled with a spline at t = 14 days was used to model rates of atrophy among type I, IIa, and IIb myofibers; bootstrapping was used to estimate standard errors. Results are presented in Table 1.

**Table 1:** Myofiber type-dependent atrophy during acute and chronic denervation.

0–14 days denervation	Δ minimum Feret diameter (μm/day)	standard error (μm/day)	95% CI	*P* (compared to type IIb)
type IIb	−3.03	0.07	−3.16, −2.89	—
IIa	−0.80	0.04	−0.90, −0.71	<0.0001
I	−1.24	0.06	−1.36, −1.13	<0.0001
**>14 days denervation**				
type IIb	−0.17	0.01	−0.19, −0.15	—
IIa	−0.11	0.01	−0.14, −0.09	<0.0001
I	−0.09	0.01	−0.11, −0.06	<0.0001

### RNA quality control

**Fig. 3:**
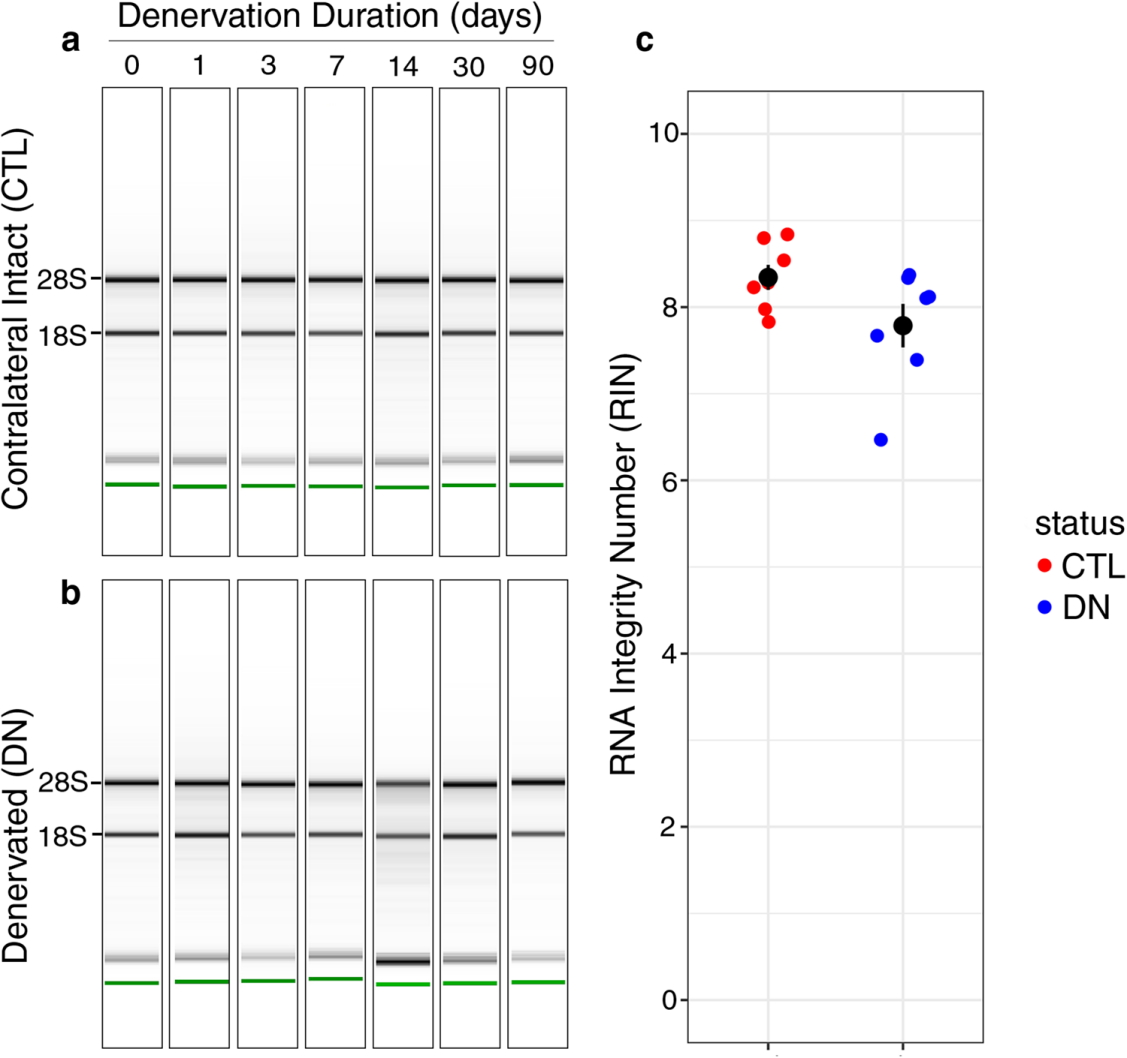
RNA integrity of samples. Following denervation for the designated durations, denervated and contralateral intact gastrocnemii were harvested and homogenized directly in TRIzol, and total RNA was column-purified. RNA samples were reverse-transcribed to cDNA and sequenced on an Illumina platform. Representative RIN tracings from one biological replicate of the cohort, showing total RNA isolated from intact gastrocnemii (**a**) and paired contralateral denervated gastrocnemii (**b**). RNA isolated from denervated and intact muscle showed similar quality (**c**).

RNA integrity was analyzed using an Agilent 2100 Bioanalyzer (Fig. 3). The mean RNA Integrity Number (RIN) for RNA isolated from denervated and contralateral intact gastrocnemii was 7.8 ± 0.3 and 8.3 ± 0.1 (mean ± SEM), respectively, with no significant difference in RIN by denervation status.

### Read quality and base-calling accuracy

**Fig. 4:**
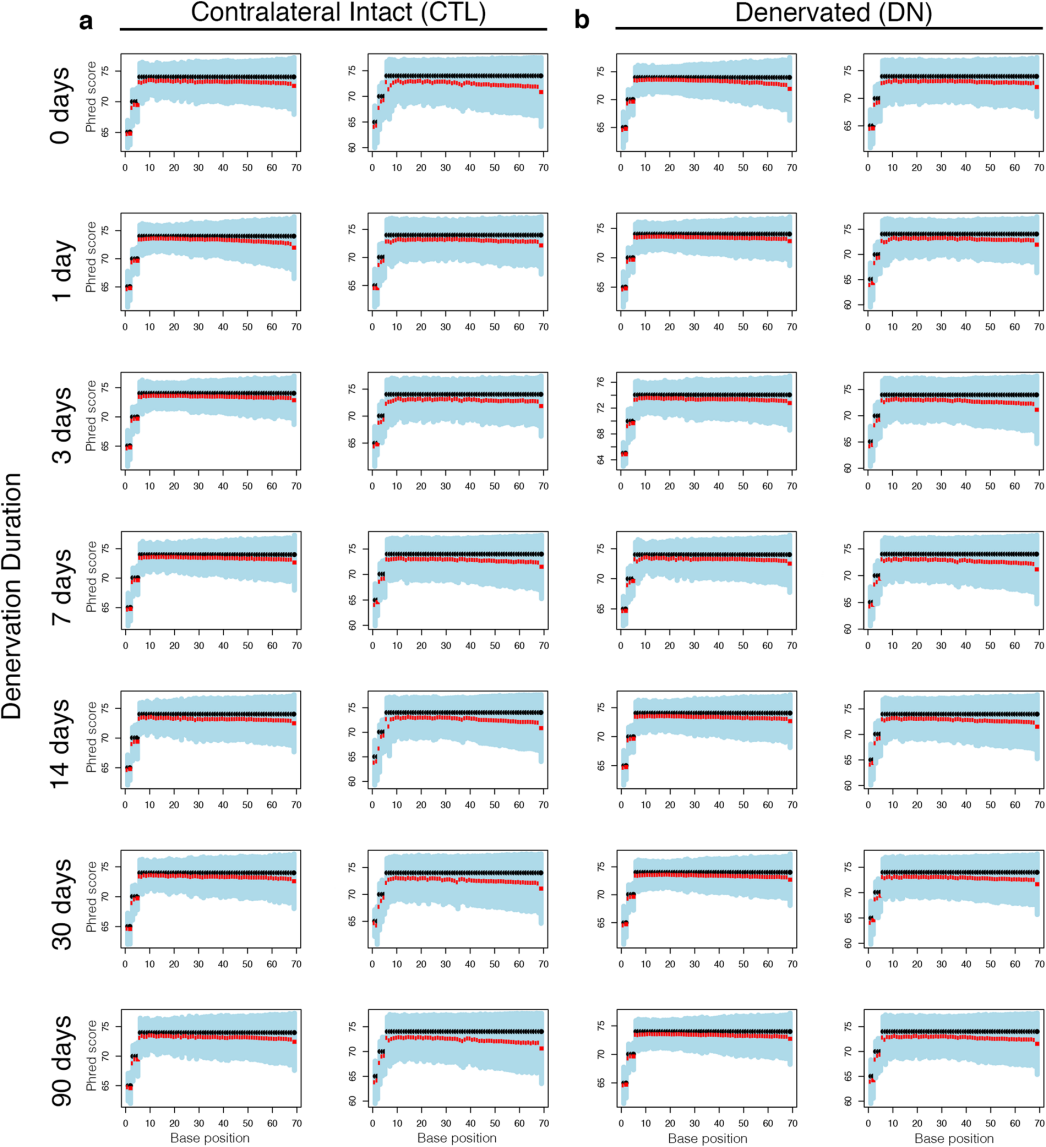
Read quality. Representative distribution of Phred quality scores at each nucleotide, shown for the paired reads of one biological replicate for contralateral intact (**a**) and denervated (**b**) muscle. The boxes indicate the mean, median, and lower and upper quartile.

**Fig. 5:**
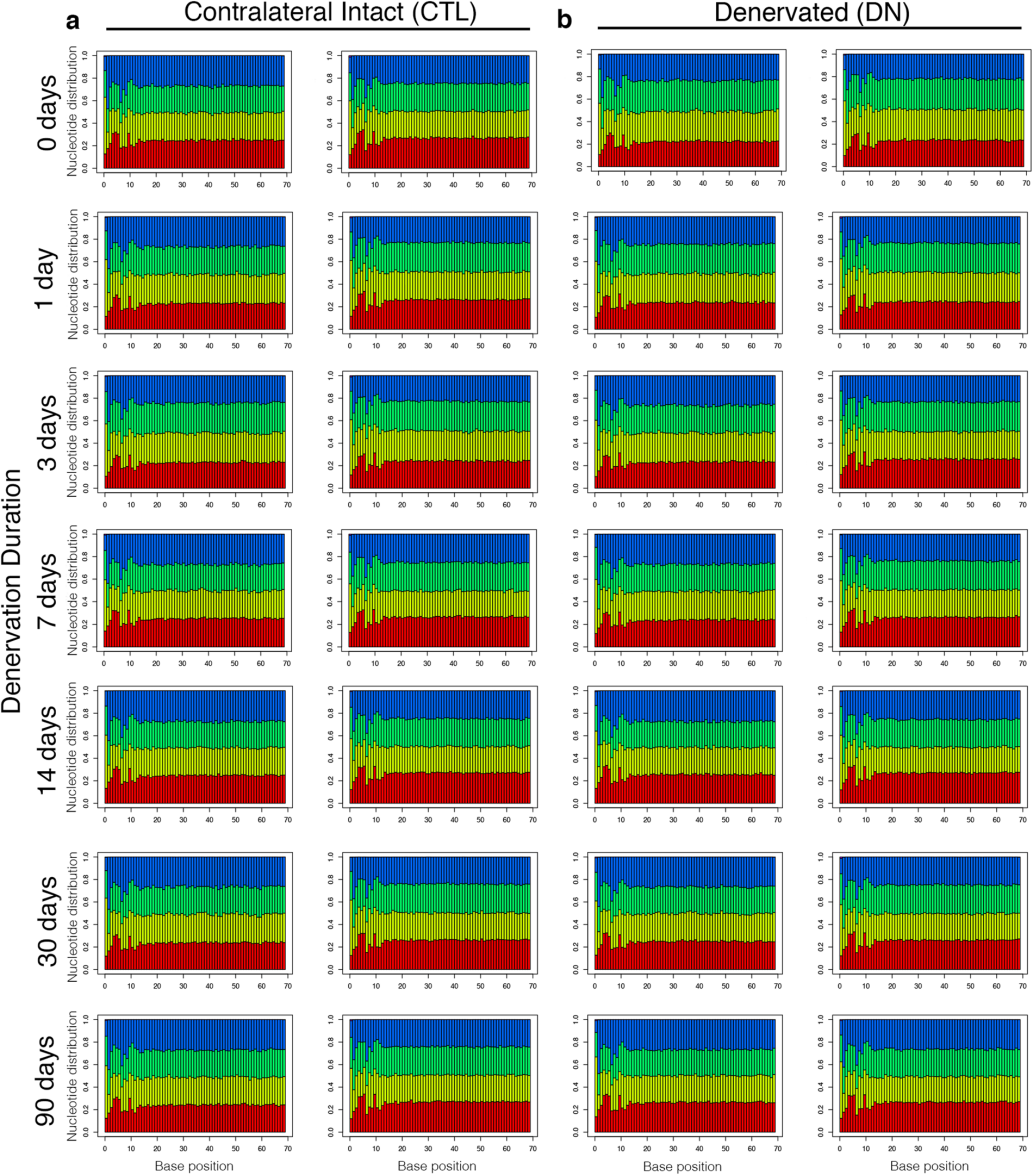
Alignment quality. Representative distribution of A (red), C (yellow), G (green), and T (blue) at each nucleotide, shown for the paired reads of one biological replicate for contralateral intact (**a**) and denervated (**b**) muscle.

Read quality was high with Phred quality score >70 for the majority of the cycles, and lower quartile base qualities were generally high (Fig. 4). No reads or samples necessitated exclusion based on read quality. The nucleotide composition patterns (proportions of A/C/G/T) of all samples were as expected, with nearly uniform proportions of each nucleotide across sequencing cycles (with the exception of a non-random pattern of nucleotide proportions in the first 13 sequencing cycles as a result of random hexamer priming) (Fig. 5). No read trimming or filtering was required because the quality distribution and variance appeared normal across all reads and samples.

### Alignment quality

A summary of alignment statistics for all samples is provided in Tables 2–9. Similar sequencing depths and mapping rates were observed for the denervated and contralateral intact skeletal muscle samples.

**Table 2:** Overall summary of alignments.

	CTL	DN	*P* (DN vs. CTL)*
	Mean (SD), range	Mean (SD), range	
Number of input reads	5.71 × 10^7^ (8.50 × 10^6^) 3.91 × 10^7^–7.75 × 10^7^	5.90 × 10^7^ (1.15 × 10^7^) 4.15 × 10^7^–9.10 × 10^7^	0.49
Average input read length	138	138	—
Number of uniquely mapped reads	4.6 × 10^7^ (7.29 × 10^6^) 3.17 × 10^7^–5.88 × 10^7^	4.89 × 10^7^ (8.40 × 10^6^) 3.57 × 10^7^–6.73 × 10^7^	0.17
Uniquely mapped reads (%)	80.47 (3.75) 73.85–85.92	83.29 (3.99) 72.3–88.62	0.009
Average mapped length	137.39 (0.42) 136.10–137.71	137.45 (0.41) 135.93–137.71	0.58
Number of splices: Total	1.62 × 10^7^ (4.93 × 10^6^) 5.67 × 10^6^–2.45 × 10^7^	1.72 × 10^7^ (3.35 × 10^6^) 1.00 × 10^7^–2.38 × 10^7^	0.37
Number of splices: Annotated (sjdb)	1.60 × 10^7^ (4.91 × 10^6^) 5.51 × 10^6^–2.43 × 10^7^	1.70 × 10^7^ (3.33 × 10^6^) 9.86 × 10^6^–2.35 × 10^7^	0.38
Number of splices: GT/AG	1.61 × 10^7^ (4.90 × 10^6^) 5.59 × 10^6^–2.44 × 10^7^	1.71 × 10^7^ (3.33 × 10^6^) 9.92 × 10^6^–2.36 × 10^7^	0.38
Number of splices: GC/AG	9.38 × 10^4^ (2.83 × 10^4^) 3.37 × 10^4^–1.42 × 10^5^	1.09 × 10^5^ (13.37 × 10^4^) 7.05 × 10^4^–1.52 × 10^5^	0.03
Number of splices: AT/AC	7.31 × 10^3^ (2.18 × 10^3^) 2.46 × 10^3^–1.13 × 10^4^	9.38 × 10^3^ (1.95 × 10^3^) 5.41 × 10^3^–1.49 × 10^4^	0.0004
Number of splices: Non-canonical	2.79 × 10^4^ (8.77 × 10^3^) 1.44 × 10^4^–4.91 × 10^4^	2.77 × 10^4^ (8.62 × 10^3^) 1.76 × 10^4^–5.24 × 10^4^	0.92
Mismatch rate per base (%)	0.28 (0.14) 0.15–0.66	0.25 (0.12) 0.15–0.68	0.32
Deletion rate per base (%)	0.002 (0.004) 0–0.01	0.001 (0.004) 0–0.01	0.49
Deletion average length	1.68 (0.32) 1.35–2.66	1.72 (0.29) 1.41–2.78	0.65
Insertion rate per base (%)	0.005 (0.008) 0–0.03	0.003 (0.007) 0–0.03	0.38
**Multi-Mapping Reads:**			
Number of reads mapped to multiple loci	7.09 × 10^6^ (1.34 × 10^6^) 4.89 × 10^6^–1.03 × 10^7^	6.32 × 10^6^ (2.01 × 10^6^) 3.27 × 10^6^–1.29 × 10^7^	0.10
% of reads mapped to multiple loci	12.46 (1.65) 9.61–15.87	10.64 (2.15) 7.26–16.89	0.0008
Number of reads mapped to too many loci	6.16 × 10^5^ (4.96 × 10^5^) 1.31 × 10^5^–1.98 × 10^6^	4.85 × 10^5^ (3.25 × 10^5^) 1.45 × 10^5^–1.66 × 10^6^	0.25
**Unmapped Reads:**			
% of reads unmapped: too many mismatches	0	0	—
% of reads unmapped: too short	5.32 (2.21) 2.99–11.82	4.71 (2.29) 2.63–13.22	0.32

^*^Welch’s t-test.

**Table 3:** Day 0 (baseline) alignments.

	CTL-0-1	CTL-0-2	CTL-0-3	CTL-0-4	DN-0-1	DN-0-2	DN-0-3	DN-0-4
Number of input reads	55,742,415	55,520,609	55,035,747	63,030,555	61,655,193	46,879,302	68,278,353	90,993,687
Average input read length	138	138	138	138	138	138	138	138
Number of uniquely mapped reads	44,288,330	42,269,606	46,785,173	52,763,039	49,365,745	36,068,973	56,573,593	65,791,821
Uniquely mapped reads (%)	79.45	76.13	85.01	83.71	80.07	76.94	82.86	72.30
Average mapped length	137.29	136.1	137.64	137.57	137.2	137.61	137.61	135.93
Number of splices: Total	13,449,628	11,337,800	17,752,380	19,213,536	13,937,303	15,460,977	23,318,214	22,103,629
Number of splices: Annotated (sjdb)	13,279,964	11,188,952	17,562,687	18,981,111	13,722,595	15,257,243	23,063,152	21,856,608
Number of splices: GT/AG	13,338,075	11,231,532	17,622,780	19,059,810	13,811,905	15,337,675	23,148,268	21,924,998
Number of splices: GC/AG	79,141	70,961	100,965	116,597	83,254	93,607	131,002	128,493
Number of splices: AT/AC	6,613	5,470	7,814	8,779	7,204	7,569	10,127	10,049
Number of splices: Non-canonical	25,799	29,837	20,821	28,350	39,940	22,126	28,817	40,089
Mismatch rate per base (%)	0.32	0.64	0.19	0.21	0.31	0.22	0.19	0.68
Deletion rate per base (%)	0.00	0.01	0.00	0.00	0.00	0.00	0.00	0.01
Deletion average length	1.84	2.66	1.55	1.59	1.65	1.46	1.56	2.78
Insertion rate per base (%)	0.01	0.03	0.00	0.00	0.01	0.00	0.00	0.03
**Multi-Mapping Reads:**								
Number of reads mapped to multiple loci	7,297,246	6,213,049	5,623,293	6,892,906	7,920,730	7,915,620	7,813,515	12,905,331
% of reads mapped to multiple loci	13.09	11.19	10.22	10.94	12.85	16.89	11.44	14.18
Number of reads mapped to too many loci	582,753	255,211	341,804	568,680	987,746	539,784	463,089	172,576
**Unmapped Reads:**								
% of reads unmapped: too many mismatches	0.00	0.00	0.00	0.00	0.00	0.00	0.00	0.00
% of reads unmapped: too short	5.63	11.82	3.69	3.95	4.63	4.62	4.66	13.22

**Table 4:** Day 1 post-denervation alignments.

	CTL-1-1	CTL-1-2	CTL-1-3	CTL-1-4	DN-1-1	DN-1-2	DN-1-3	DN-1-4
Number of input reads	61,636,872	58,072,077	55,973,096	71,794,344	58,034,876	48,989,931	73,886,622	74,271,951
Average input read length	138	138	138	138	138	138	138	138
Number of uniquely mapped reads	45,746,325	47,654,856	48,094,866	58,843,890	48,750,120	39,908,061	62,480,235	56,047,037
Uniquely mapped reads (%)	74.22	82.06	85.92	81.96	84.00	81.46	84.56	75.46
Average mapped length	137.34	137.47	137.57	137.6	137.62	137.59	137.57	136.25
Number of splices: Total	9,070,911	16,321,200	18,716,287	24,549,738	17,994,490	13,613,414	23,080,273	17,817,564
Number of splices: Annotated (sjdb)	8,836,384	16,122,233	18,519,783	24,297,361	17,785,158	13,408,135	22,808,431	17,582,261
Number of splices: GT/AG	8,966,851	16,190,887	18,583,614	24,365,743	17,858,255	13,494,722	22,900,176	17,663,375
Number of splices: GC/AG	50,713	96,907	103,605	141,926	103,877	83,585	134,880	108,178
Number of splices: AT/AC	4,255	7,466	8,267	11,276	8,327	6,878	11,030	8,758
Number of splices: Non-canonical	49,092	25,940	20,801	30,793	24,031	28,179	34,187	37,253
Mismatch rate per base (%)	0.40	0.26	0.23	0.17	0.19	0.25	0.21	0.63
Deletion rate per base	0.01	0.00	0.00	0.00	0.00	0.00	0.00	0.01
Deletion average length	1.37	1.63	1.84	1.71	1.49	1.45	1.66	2.4
Insertion rate per base	0.00	0.01	0.00	0.01	0.00	0.00	0.00	0.02
**Multi-Mapping Reads:**								
Number of reads mapped to multiple loci	9,782,705	6,777,446	5,377,772	8,642,605	6,176,521	5,844,014	7,396,719	9,668,787
% of reads mapped to multiple loci	15.87	11.67	9.61	12.04	10.64	11.93	10.01	13.02
Number of reads mapped to too many loci	1,984,894	619,936	341,393	194,108	530,916	812,052	596,417	413,918
**Unmapped Reads:**								
% of reads unmapped: too many mismatches	0.00	0.00	0.00	0.00	0.00	0.00	0.00	0.00
% of reads unmapped: too short	5.12	4.55	3.44	5.55	3.79	4.17	4.13	10.71

**Table 5:** Day 3 post-denervation alignments.

	CTL-3-1	CTL-3-2	CTL-3-3	CTL-3-4	DN-3-1	DN-3-2	DN-3-3	DN-3-4
Number of input reads	43,601,767	39,051,346	60,982,532	77,529,167	61,614,609	48,980,170	77,233,032	49,001,916
Average input read length	138	138	138	138	138	138	138	138
Number of uniquely mapped reads	32,795,169	31,708,280	51,656,264	58,276,505	48,300,398	40,533,615	67,261,853	40,543,472
Uniquely mapped reads (%)	75.22	81.20	84.71	75.17	78.39	82.76	87.09	82.74
Average mapped length	137.18	137.54	137.63	136.29	137.27	137.61	137.58	137.7
Number of splices: Total	7,641,732	14,023,149	19,917,676	20,730,824	12,188,893	15,689,660	23,843,242	18,125,528
Number of splices: Annotated (sjdb)	7,481,956	13,875,662	19,704,844	20,503,659	11,912,730	15,485,355	23,536,888	17,920,443
Number of splices: GT/AG	7,559,269	13,909,110	19,774,059	20,569,914	12,051,290	15,551,272	23,642,787	17,982,044
Number of splices: GC/AG	44,922	89,610	110,632	117,092	76,969	104,920	151,795	114,053
Number of splices: AT/AC	3,751	6,827	8,919	9,864	8,217	10,333	14,859	11,510
Number of splices: Non-canonical	33,790	17,602	24,066	33,954	52,417	23,135	33,801	17,921
Mismatch rate per base (%)	0.37	0.18	0.18	0.57	0.35	0.20	0.21	0.15
Deletion rate per base	0.00	0.00	0.00	0.01	0.01	0.00	0.00	0.00
Deletion average length	1.45	1.93	1.54	2.64	1.41	1.76	1.74	1.65
Insertion rate per base	0.00	0.01	0.00	0.02	0.00	0.00	0.00	0.00
**Multi-Mapping Reads:**								
Number of reads mapped to multiple loci	5,971,134	4,893,812	6,531,891	10,323,333	8,224,640	5,219,448	6,412,447	6,047,917
% of reads mapped to multiple loci	13.69	12.53	10.71	13.32	13.35	10.66	8.30	12.34
Number of reads mapped to too many loci	1,070,563	150,149	453,705	168,949	1,656,313	391,356	516,587	143,641
**Unmapped Reads:**								
% of reads unmapped: too many mismatches	0.00	0.00	0.00	0.00	0.00	0.00	0.00	0.00
% of reads unmapped: too short	7.35	5.56	3.28	11.17	4.33	5.33	3.35	4.41

**Table 6:** Day 7 post-denervation alignments.

	CTL-7-1	CTL-7-2	CTL-7-3	CTL-7-4	DN-7-1	DN-7-2	DN-7-3	DN-7-4
Number of input reads	60,590,610	56,143,558	52,019,275	51,496,201	59,955,862	52,135,014	56,347,860	49,238,405
Average input read length	138	138	138	138	138	138	138	138
Number of uniquely mapped reads	44,746,349	45,413,965	43,059,558	41,428,228	48,787,077	44,968,959	48,225,333	41,382,042
Uniquely mapped reads (%)	73.85	80.89	82.78	80.45	81.37	86.25	85.59	84.04
Average mapped length	137.59	137.52	137.55	137.52	137.54	137.62	137.62	137.71
Number of splices: Total	14,703,980	16,917,306	15,994,824	19,253,856	18,108,367	17,195,995	19,296,266	17,537,132
Number of splices: Annotated (sjdb)	14,509,688	16,698,117	15,827,806	19,075,521	17,848,555	16,979,726	19,078,139	17,331,026
Number of splices: GT/AG	14,581,736	16,782,958	15,879,431	19,119,493	17,951,606	17,055,436	19,144,656	17,402,247
Number of splices: GC/AG	87,629	94,175	90,057	109,413	113,654	107,871	121,143	107,129
Number of splices: AT/AC	6,982	7,259	6,713	8,335	11,444	10,331	10,951	10,114
Number of splices: Non-canonical	27,633	32,914	18,623	16,615	31,663	22,357	19,516	17,642
Mismatch rate per base (%)	0.23	0.25	0.24	0.20	0.21	0.19	0.23	0.16
Deletion rate per base	0.00	0.00	0.00	0.00	0.00	0.00	0.00	0.00
Deletion average length	1.46	1.6	1.55	1.66	1.5	1.68	1.61	1.58
Insertion rate per base	0.00	0.00	0.00	0.01	0.00	0.00	0.00	0.00
**Multi-Mapping Reads**:								
Number of reads mapped to multiple loci	9,428,246	6,672,205	5,740,042	6,966,763	7,352,327	4,543,202	5,722,848	5,194,049
% of reads mapped to multiple loci	15.56	11.88	11.03	13.53	12.26	8.71	10.16	10.55
Number of reads mapped to too many loci	819,014	789,365	391,842	166,663	716,324	332,671	233,636	243,367
**Unmapped Reads:**								
% of reads unmapped: too many mismatches	0.00	0.00	0.00	0.00	0.00	0.00	0.00	0.00
% of reads unmapped: too short	8.41	5.23	4.83	5.53	4.51	3.91	3.51	4.58

**Table 7:** Day 14 post-denervation alignments.

	CTL-14-1	CTL-14-2	CTL-14-3	CTL-14-4	DN-14-1	DN-14-2	DN-14-3	DN-14-4
Number of input reads	49,441,646	69,876,924	57,832,497	47,973,299	55,891,429	44,966,509	59,715,016	54,942,368
Average input read length	138	138	138	138	138	138	138	138
Number of uniquely mapped reads	36,913,926	57,899,595	48,950,623	38,979,826	46,402,585	39,850,074	51,086,876	47,212,433
Uniquely mapped reads (%)	74.66	82.86	84.64	81.25	83.02	88.62	85.55	85.93
Average mapped length	136.42	137.57	137.64	137.71	137.43	137.6	137.26	137.64
Number of splices: Total	5,674,228	21,316,991	17,945,548	18,202,915	14,603,778	13,578,938	17,163,851	19,981,473
Number of splices: Annotated (sjdb)	5,507,058	21,070,682	17,756,782	18,029,158	14,378,276	13,404,598	16,946,761	19,745,905
Number of splices: GT/AG	5,594,717	21,157,480	17,811,041	18,071,861	14,465,582	13,467,591	17,016,218	19,822,262
Number of splices: GC/AG	33,738	117,529	105,892	108,309	97,389	86,275	114,337	126,876
Number of splices: AT/AC	2,460	8,933	7,681	8,376	8,415	7,229	9,540	11,690
Number of splices: Non-canonical	43,313	33,049	20,934	14,369	32,392	17,843	23,756	20,645
Mismatch rate per base (%)	0.66	0.22	0.19	0.15	0.23	0.21	0.30	0.17
Deletion rate per base	0.01	0.00	0.00	0.00	0.00	0.00	0.00	0.00
Deletion average length	1.96	1.53	1.49	1.54	1.6	1.73	1.77	1.81
Insertion rate per base	0.02	0.00	0.00	0.00	0.00	0.00	0.00	0.00
**Multi-Mapping Reads:**								
Number of reads mapped to multiple loci	6,729,802	8,099,802	6,448,816	6,968,357	5,711,963	3,266,255	5,086,857	5,109,283
% of reads mapped to multiple loci	13.61	11.59	11.15	14.53	10.22	7.26	8.52	9.30
Number of reads mapped to too many loci	1,288,797	704,083	369,246	131,113	711,573	285,356	277,105	152,218
**Unmapped Reads:**								
% of reads unmapped: too many mismatches	0.00	0.00	0.00	0.00	0.00	0.00	0.00	0.00
% of reads unmapped: too short	7.92	3.98	2.99	3.77	4.72	2.93	4.97	4.27

**Table 8:** Day 30 post-denervation alignments.

	CTL-30-1	CTL-30-2	CTL-30-3	CTL-30-4	DN-30-1	DN-30-2	DN-30-3	DN-30-4
Number of input reads	52,742,878	46,463,403	57,501,219	60,468,553	73,590,727	48,399,322	51,665,579	54,577,655
Average input read length	138	138	138	138	138	138	138	138
Number of uniquely mapped reads	40,308,924	38,146,896	48,521,247	49,438,872	58,520,852	41,879,303	45,337,494	46,751,912
Uniquely mapped reads (%)	76.43	82.10	84.38	81.76	79.52	86.53	87.75	85.66
Average mapped length	137.26	137.46	137.68	137.65	137.6	137.5	137.65	137.55
Number of splices: Total	9,002,164	12,162,162	17,453,824	21,318,005	19,800,202	14,238,822	17,245,138	17,175,362
Number of splices: Annotated (sjdb)	8,779,541	11,999,102	17,264,634	21,090,387	19,528,567	14,056,750	17,044,059	16,948,110
Number of splices: GT/AG	8,902,807	12,063,473	17,319,770	21,157,409	19,623,217	14,122,315	17,105,745	17,027,539
Number of splices: GC/AG	50,013	69,863	105,703	125,956	132,947	88,561	112,478	111,959
Number of splices: AT/AC	4,129	5,439	7,989	9,587	11,133	6,876	8,957	9,017
Number of splices: Non-canonical	45,215	23,387	20,362	25,053	32,905	21,070	17,958	26,847
Mismatch rate per base (%)	0.41	0.34	0.18	0.18	0.21	0.23	0.16	0.22
Deletion rate per base	0.01	0.00	0.00	0.00	0.00	0.00	0.00	0.00
Deletion average length	1.35	1.54	1.52	1.54	1.59	2.1	1.61	1.85
Insertion rate per base	0.00	0.00	0.00	0.00	0.00	0.01	0.00	0.01
**Multi-Mapping Reads:**								
Number of reads mapped to multiple loci	7,401,284	5,478,248	6,190,600	8,046,293	8,580,950	4,181,432	4,632,180	5,281,885
% of reads mapped to multiple loci	14.03	11.79	10.77	13.31	11.66	8.64	8.97	9.68
Number of reads mapped to too many loci	1,771,867	704,627	353,874	396,886	672,558	312,239	158,758	369,051
**Unmapped Reads:**								
% of reads unmapped: too many mismatches	0.00	0.00	0.00	0.00	0.00	0.00	0.00	0.00
% of reads unmapped: too short	4.84	3.82	3.70	3.98	7.27	3.68	2.63	3.59

**Table 9:** Day 90 post-denervation alignments.

	CTL-90-1	CTL-90-2	CTL-90-3	CTL-90-4	DN-90-1	DN-90-2	DN-90-3	DN-90-4
Number of input reads	60,873,629	47,878,309	64,409,589	64,474,030	67,574,028	41,457,417	56,366,231	64,290,975
Average input read length	138	138	138	138	138	138	138	138
Number of uniquely mapped reads	48,591,921	36,558,406	54,742,091	52,310,648	55,842,215	35,688,427	49,296,566	56,228,095
Uniquely mapped reads (%)	79.82	76.36	84.99	81.13	82.64	86.08	87.46	87.46
Average mapped length	137.54	137.33	137.65	137.54	137.46	137.62	137.69	137.56
Number of splices: Total	17,976,086	9,096,031	19,807,129	23,581,453	14,738,624	10,009,836	16,531,735	17,148,016
Number of splices: Annotated (sjdb)	17,769,205	8,892,794	19,598,023	23,349,415	14,498,430	9,862,716	16,325,792	16,910,940
Number of splices: GT/AG	17,834,684	9,003,295	19,661,022	23,405,840	14,592,638	9,915,222	16,382,464	16,990,053
Number of splices: GC/AG	108,126	48,499	114,242	134,946	101,005	70,463	117,425	118,687
Number of splices: AT/AC	8,761	3,781	8,574	10,432	7,968	5,410	9,247	9,501
Number of splices: Non-canonical	24,515	40,456	23,291	30,235	37,013	18,741	22,599	29,775
Mismatch rate per base (%)	0.20	0.41	0.19	0.19	0.25	0.21	0.17	0.22
Deletion rate per base	0.00	0.01	0.00	0.00	0.01	0.00	0.00	0.00
Deletion average length	1.75	1.37	1.52	1.96	1.61	1.55	1.67	1.88
Insertion rate per base	0.01	0.00	0.00	0.01	0.01	0.00	0.00	0.00
**Multi-Mapping Reads:**								
Number of reads mapped to multiple loci	7,924,888	7,093,313	6,681,303	8,439,457	6,752,769	3,853,430	5,037,828	5,238,591
% of reads mapped to multiple loci	13.02	14.82	10.37	13.09	9.99	9.29	8.94	8.15
Number of reads mapped to too many loci	469,250	1,545,968	447,456	176,878	855,527	419,702	172,340	444,439
**Unmapped Reads:**								
% of reads unmapped: too many mismatches	0.00	0.00	0.00	0.00	0.00	0.00	0.00	0.00
% of reads unmapped: too short	5.92	4.16	3.34	5.34	5.23	2.77	2.88	3.10

### Counts per gene

**Fig. 6:**
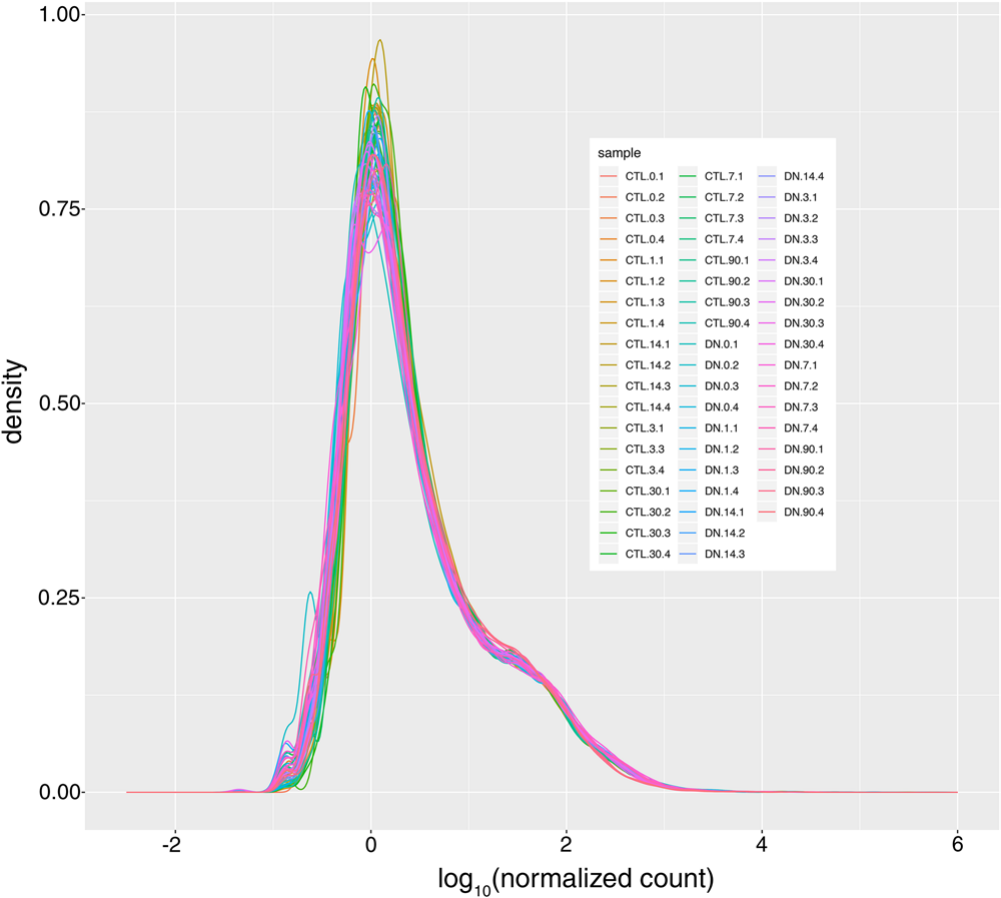
Summary of read counts. Density plot showing relative read count distributions for all samples.

The distribution of normalized gene accounts appears similar among all samples in the dataset (Fig. 6).

### Unsupervised clustering analysis of longitudinally denervated samples

**Fig. 7:**
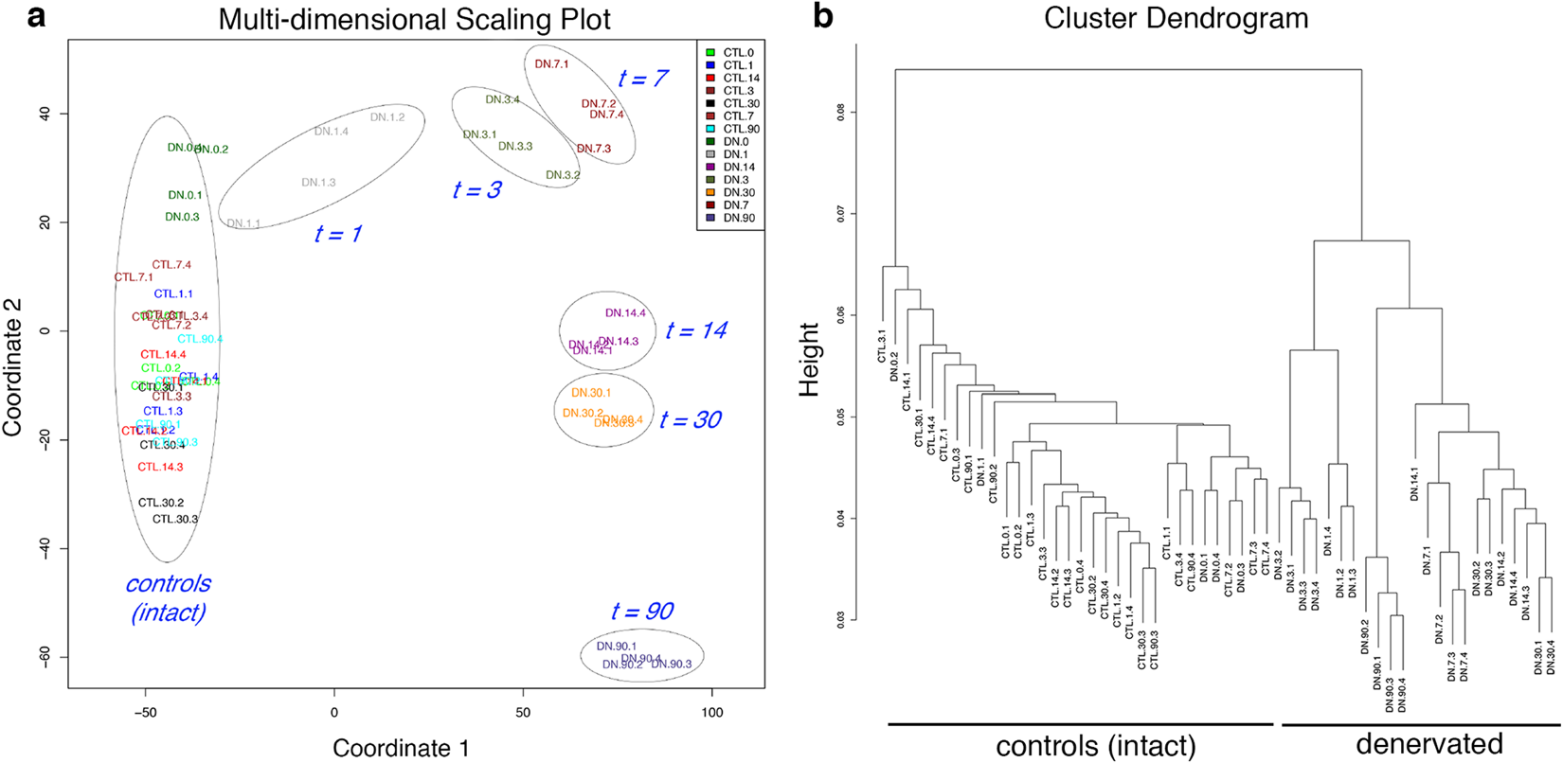
Quality of replicates. Multi-dimensional scaling analysis (**a**) and cluster dendrogram (**b**) of transcriptional profiles during neurogenic atrophy shows temporal clustering by denervation status.

Multidimensional scaling using expression levels of all genes demonstrated temporal clustering based on denervation status, with replicates within each denervation timepoint clustering closer to each other than to other denervation timepoints (Fig. 7).

### Time-dependent comparison of denervated and contralateral intact skeletal muscle transcriptomes

**Fig. 8:**
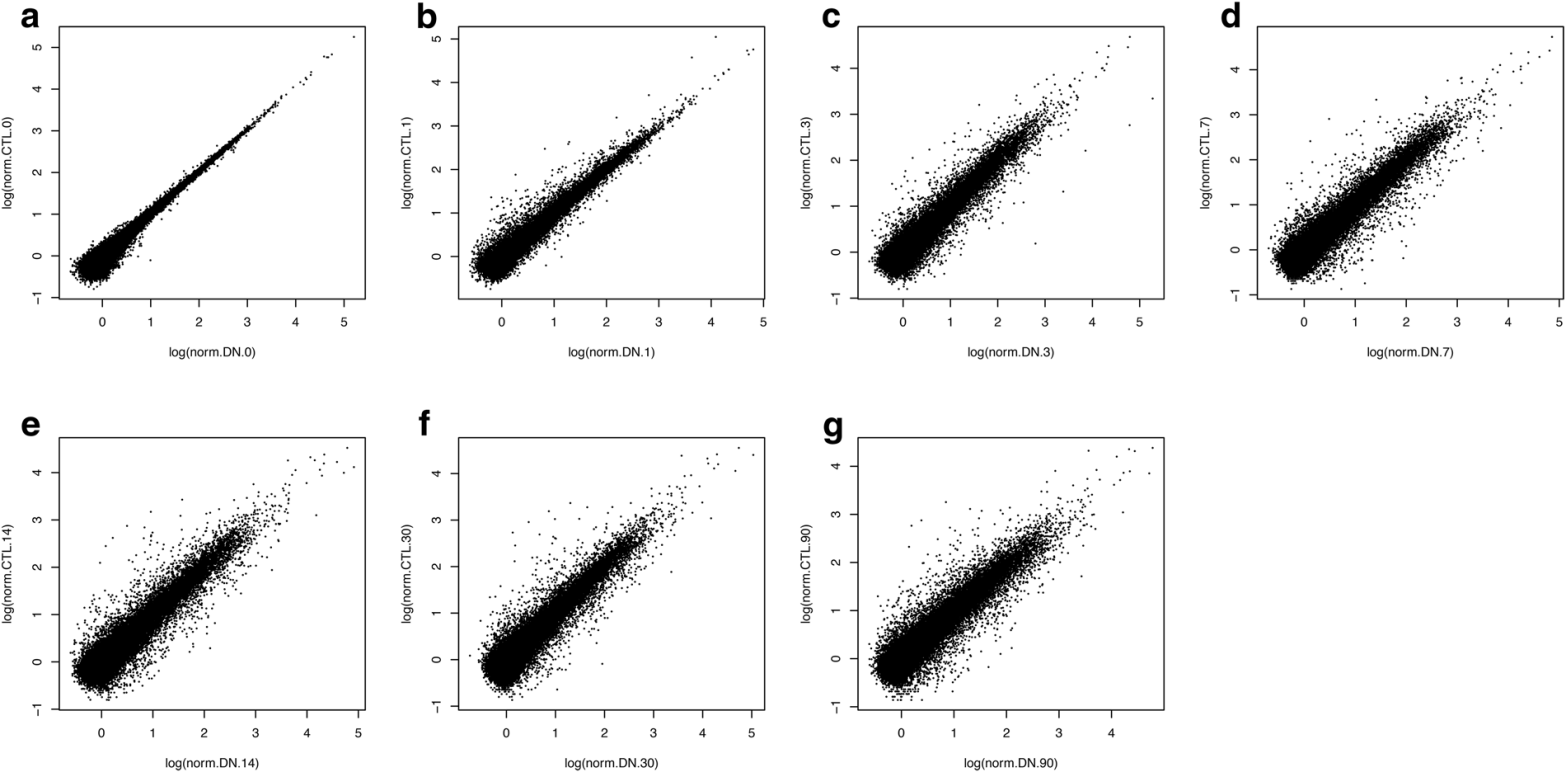
Gene expression visualization. Scatterplots showing the log2 transform of normalized counts.

Normalized gene counts from denervated and contralateral intact skeletal muscle at each timepoint are compared in scatter plots (Fig. 8).

### Differential expression analysis

**Fig. 9:**
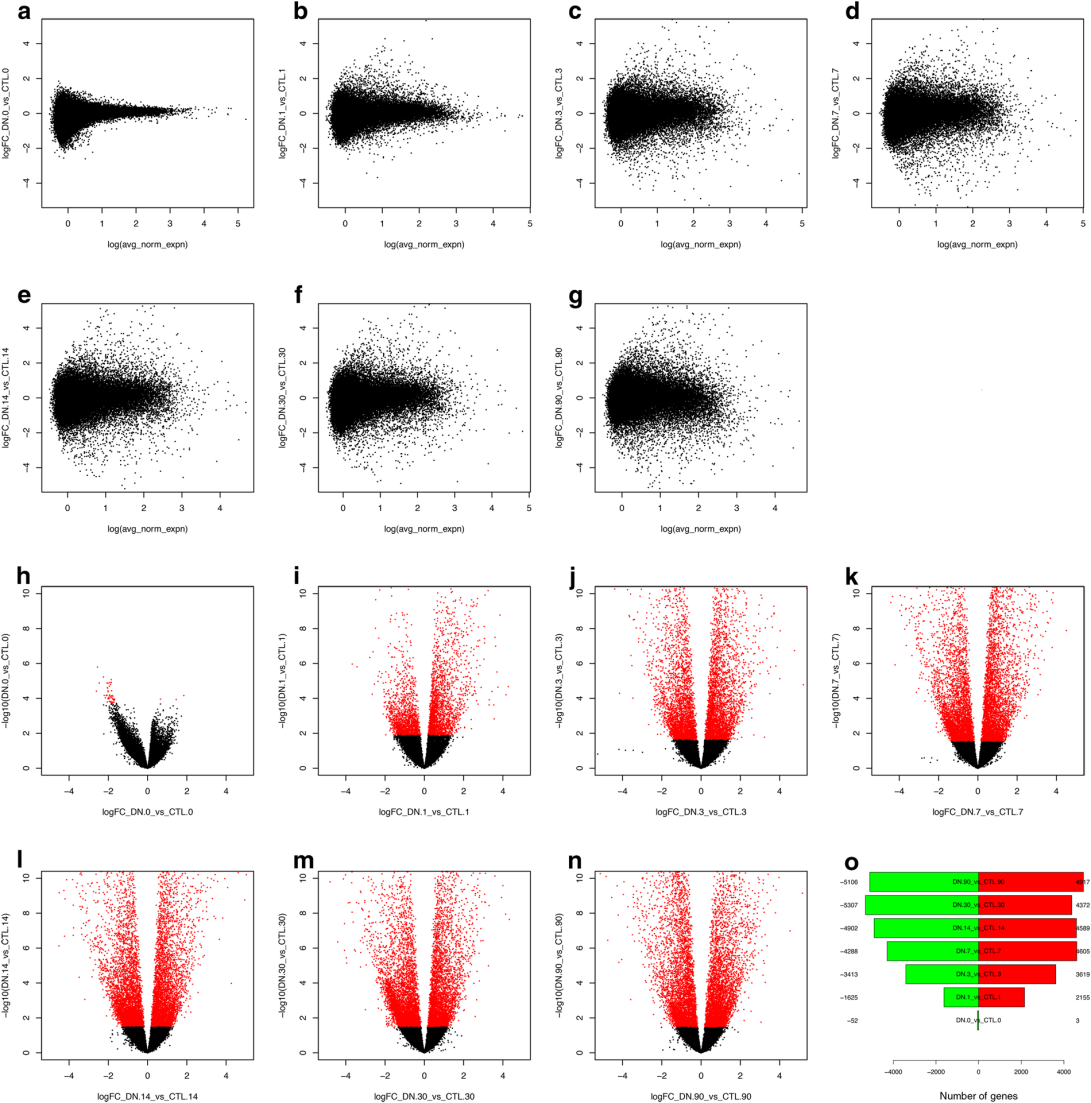
Differential expression analysis. MA-plots comparing the log2 fold change of gene expression for denervated vs. contralateral intact skeletal muscle at each timepoint plotted against the normalized average of the counts (**a**–**g**). Volcano plots showing the -log10 FDR for difference in expression between denervated and contralateral intact skeletal muscle for each gene detected, plotted against the log2 fold-change (**h**–**n**). Genes with FDR < 0.1 are depicted in red. The total number of significantly differentially expressed genes (FDR < 0.1) at each timepoint is summarized in panel (o).

MA-plots showing the log-fold change (M-values, the log of the ratio of counts for each gene across the two samples being compared) against the normalized log-average (A-values, the average counts for each gene across the two samples being compared) indicate substantial differences in gene expression in skeletal muscle during acute and chronic neurogenic atrophy (Fig. 9a–g). Volcano plots indicate minimal differences in gene expression at baseline (intact muscle) (Fig. 9h), but demonstrate that thousands of genes are significantly differentially expressed (FDR < 0.1) within the first day after denervation (Fig. 9i) and beyond (Fig. 9j–n). A summary of the number of differentially expressed genes at each timepoint is shown in Fig. 9o.

## Usage Notes

The RNA-Seq dataset presented in this study provides a detailed view of the acute and chronic gene expression changes that occur in denervated, atrophying skeletal muscle. These data may provide insight into the early events associated with acute loss of neuronal input that trigger rapid atrophy, as well as the gene expression changes in chronically denervated and severely atrophied skeletal muscle associated with impaired capacity for reinnervation. Defining these changes may afford opportunities to limit the rate and severity of skeletal muscle atrophy, and to enhance functional reinnervation.

## Supplementary Information

Supplementary Table 1

## Data Availability

Scripts used in the RNA sequencing analyses are available at https://github.com/icnn/RNAseq-PIPELINE.git.
