# Supplementary material for: Longitudinal RNA-Seq analysis of acute and chronic neurogenic skeletal muscle atrophy
Source: Sci Data. 2019 Sep 24;6:179. doi: 10.1038/s41597-019-0185-4 (PMC6760191; doi:10.1038/s41597-019-0185-4)
Supplement: Supplementary file 1 — Supplementary Table 1 [file 41597_2019_185_MOESM1_ESM.pdf]

Table S1. Metadata of all sequenced samples comprising the dataset.

Sample ID	Status	Gender	Age (months)	Denervation Duration (days)	SRAsampleID	Fastq file datalink
CTL-0-1	CTL	male	5	0	SAMN11603134	https://www.ncbi.nlm.nih.gov/biosample/11603134
CTL-0-2	CTL	male	5	0	SAMN11603135	https://www.ncbi.nlm.nih.gov/biosample/11603135
CTL-0-3	CTL	male	5	0	SAMN11603136	https://www.ncbi.nlm.nih.gov/biosample/11603136
CTL-0-4	CTL	male	5	0	SAMN11603137	https://www.ncbi.nlm.nih.gov/biosample/11603137
CTL-1-1	CTL	male	5	1	SAMN11603138	https://www.ncbi.nlm.nih.gov/biosample/11603138
CTL-1-2	CTL	male	5	1	SAMN11603139	https://www.ncbi.nlm.nih.gov/biosample/11603139
CTL-1-3	CTL	male	5	1	SAMN11603140	https://www.ncbi.nlm.nih.gov/biosample/11603140
CTL-1-4	CTL	male	5	1	SAMN11603141	https://www.ncbi.nlm.nih.gov/biosample/11603141
CTL-3-1	CTL	male	5	3	SAMN11603142	https://www.ncbi.nlm.nih.gov/biosample/11603142
CTL-3-2	CTL	male	5	3	SAMN11603143	https://www.ncbi.nlm.nih.gov/biosample/11603143
CTL-3-3	CTL	male	5	3	SAMN11603144	https://www.ncbi.nlm.nih.gov/biosample/11603144
CTL-3-4	CTL	male	5	3	SAMN11603145	https://www.ncbi.nlm.nih.gov/biosample/11603145
CTL-7-1	CTL	male	5	7	SAMN11603146	https://www.ncbi.nlm.nih.gov/biosample/11603146
CTL-7-2	CTL	male	5	7	SAMN11603147	https://www.ncbi.nlm.nih.gov/biosample/11603147
CTL-7-3	CTL	male	5	7	SAMN11603148	https://www.ncbi.nlm.nih.gov/biosample/11603148
CTL-7-4	CTL	male	5	7	SAMN11603149	https://www.ncbi.nlm.nih.gov/biosample/11603149
CTL-14-1	CTL	male	5	14	SAMN11603150	https://www.ncbi.nlm.nih.gov/biosample/11603150
CTL-14-2	CTL	male	5	14	SAMN11603151	https://www.ncbi.nlm.nih.gov/biosample/11603151
CTL-14-3	CTL	male	5	14	SAMN11603152	https://www.ncbi.nlm.nih.gov/biosample/11603152
CTL-14-4	CTL	male	5	14	SAMN11603153	https://www.ncbi.nlm.nih.gov/biosample/11603153
CTL-30-1	CTL	male	5	30	SAMN11603154	https://www.ncbi.nlm.nih.gov/biosample/11603154
CTL-30-2	CTL	male	5	30	SAMN11603155	https://www.ncbi.nlm.nih.gov/biosample/11603155
CTL-30-3	CTL	male	5	30	SAMN11603156	https://www.ncbi.nlm.nih.gov/biosample/11603156
CTL-30-4	CTL	male	5	30	SAMN11603157	https://www.ncbi.nlm.nih.gov/biosample/11603157
CTL-90-1	CTL	male	5	90	SAMN11603158	https://www.ncbi.nlm.nih.gov/biosample/11603158
CTL-90-2	CTL	male	5	90	SAMN11603159	https://www.ncbi.nlm.nih.gov/biosample/11603159
CTL-90-3	CTL	male	5	90	SAMN11603160	https://www.ncbi.nlm.nih.gov/biosample/11603160
CTL-90-4	CTL	male	5	90	SAMN11603161	https://www.ncbi.nlm.nih.gov/biosample/11603161
DN-0-1	DN	male	5	0	SAMN11603162	https://www.ncbi.nlm.nih.gov/biosample/11603162
DN-0-2	DN	male	5	0	SAMN11603163	https://www.ncbi.nlm.nih.gov/biosample/11603163
DN-0-3	DN	male	5	0	SAMN11603164	https://www.ncbi.nlm.nih.gov/biosample/11603164
DN-0-4	DN	male	5	0	SAMN11603165	https://www.ncbi.nlm.nih.gov/biosample/11603165
DN-1-1	DN	male	5	1	SAMN11603166	https://www.ncbi.nlm.nih.gov/biosample/11603166
DN-1-2	DN	male	5	1	SAMN11603167	https://www.ncbi.nlm.nih.gov/biosample/11603167
DN-1-3	DN	male	5	1	SAMN11603168	https://www.ncbi.nlm.nih.gov/biosample/11603168
DN-1-4	DN	male	5	1	SAMN11603169	https://www.ncbi.nlm.nih.gov/biosample/11603169
DN-3-1	DN	male	5	3	SAMN11603170	https://www.ncbi.nlm.nih.gov/biosample/11603170
DN-3-2	DN	male	5	3	SAMN11603171	https://www.ncbi.nlm.nih.gov/biosample/11603171
DN-3-3	DN	male	5	3	SAMN11603172	https://www.ncbi.nlm.nih.gov/biosample/11603172
DN-3-4	DN	male	5	3	SAMN11603173	https://www.ncbi.nlm.nih.gov/biosample/11603173
DN-7-1	DN	male	5	7	SAMN11603174	https://www.ncbi.nlm.nih.gov/biosample/11603174
DN-7-2	DN	male	5	7	SAMN11603175	https://www.ncbi.nlm.nih.gov/biosample/11603175
DN-7-3	DN	male	5	7	SAMN11603176	https://www.ncbi.nlm.nih.gov/biosample/11603176
DN-7-4	DN	male	5	7	SAMN11603177	https://www.ncbi.nlm.nih.gov/biosample/11603177
DN-14-1	DN	male	5	14	SAMN11603178	https://www.ncbi.nlm.nih.gov/biosample/11603178
DN-14-2	DN	male	5	14	SAMN11603179	https://www.ncbi.nlm.nih.gov/biosample/11603179
DN-14-3	DN	male	5	14	SAMN11603180	https://www.ncbi.nlm.nih.gov/biosample/11603180
DN-14-4	DN	male	5	14	SAMN11603181	https://www.ncbi.nlm.nih.gov/biosample/11603181
DN-30-1	DN	male	5	30	SAMN11603182	https://www.ncbi.nlm.nih.gov/biosample/11603182
DN-30-2	DN	male	5	30	SAMN11603183	https://www.ncbi.nlm.nih.gov/biosample/11603183
DN-30-3	DN	male	5	30	SAMN11603184	https://www.ncbi.nlm.nih.gov/biosample/11603184
DN-30-4	DN	male	5	30	SAMN11603185	https://www.ncbi.nlm.nih.gov/biosample/11603185
DN-90-1	DN	male	5	90	SAMN11603186	https://www.ncbi.nlm.nih.gov/biosample/11603186
DN-90-2	DN	male	5	90	SAMN11603187	https://www.ncbi.nlm.nih.gov/biosample/11603187

DN-90-3	DN	male	5	90	SAMN11603188	https://www.ncbi.nlm.nih.gov/biosample/11603188
DN-90-4	DN	male	5	90	SAMN11603189	https://www.ncbi.nlm.nih.gov/biosample/11603189
